# A bibliometric analysis of research on the treatment of facial nerve palsy

**DOI:** 10.1097/MD.0000000000026984

**Published:** 2021-08-20

**Authors:** Bonhyuk Goo, Ha-Na Kim, Jung-Hyun Kim, Sang-Soo Nam

**Affiliations:** a Department of Acupuncture & Moxibustion, Kyung Hee University Hospital at Gangdong, Gangdong-gu; b Department of Clinical Korean Medicine, Graduate School, Kyung Hee University, Dongdaemun-gu; c Department of Acupuncture & Moxibustion, College of Korean Medicine, Kyung Hee University, Dongdaemun-gu, Seoul, Republic of Korea.

**Keywords:** bibliometric analysis, complementary and alternative treatment, facial nerve palsy, medication, visualized analysis

## Abstract

**Background:**

There are various treatments for facial nerve palsy, and research into this topic is ongoing. In the present study, we carried out bibliometric and visualized analyses to identify the trends of research into facial nerve palsy treatment.

**Methods:**

To identify articles, the SCOPUS database was searched for articles published from its inception to December 27, 2020. The search was conducted twice, with Search 1 investigating general treatment trends and Search 2 narrowing the scope to complementary and alternative treatment. The extracted keywords were analyzed using the Visualization Of Similarities (VOS) viewer. Through analysis of keywords, research hotspots in the treatment of facial nerve palsy were identified.

**Results:**

A total of 1609 and 223 articles were identified in Searches 1 and 2, respectively. The number of articles published each year showed a tendency to increase, and most of the studies were only conducted in a few countries. In terms of subject area, “medicine” was overwhelmingly the most common(77.6%). Based on the analysis of 316 keywords in Search1, “medication treatment,” and “complementary and alternative treatment” were the hotspots of research.

**Conclusion:**

This study provides the overall trends of facial nerve palsy treatment. To date, research on medication treatment has been main focus, and antiviral use among medication treatment and complementary and alternative treatment has emerged in recent years.

## 1 Introduction

Facial nerve palsy is caused by acute damage to the facial nerve, most of which is idiopathic or caused by inflammation, although there are many other causes, such as trauma, space-occupying lesions, and autoimmune disease.^[1,2]^ Facial nerve palsy manifests as sudden, unilateral facial muscle weakness, causing an inability to close the eye completely, drooping of the mouth, and other symptoms such as taste disorder, hyperacusis, and dacryorrhea.^[3,4]^

Approximately 70% of patients are treated within 6 months, but the remaining 30% are fail to be fully treated, resulting in sequelae such as facial disfigurement, contracture, and synkinesis.^[5,6]^ Slow recovery and sequelae of palsy can limit social functioning and quality of life; as a result, patients experience various psychological problems.^[7–9]^ In many cases, during the early stages of the onset, patients experience fear and anxiety and gradually show signs of depression.^[10]^

The present treatment guidelines suggest using steroids and antivirals,.^[6,11]^ whereas traditional Korean medicine guidelines suggest various treatments, including acupuncture, pharmaco-acupuncture, and herbal medicine.^[12,13]^ The primary aim of facial nerve palsy treatment is rapid recovery of function, whereas the secondary aims include complete recovery without sequelae and psychological support for patients.^[1,14]^

There is a bibliometric analysis study of the treatment for facial nerve palsy that already been undertaken,.^[15]^ but this has focused only on physiotherapy and does not include the latest articles.

The purpose of this bibliometric analysis study is to investigate what treatments have been studied in the treatment of facial nerve palsy to date and ascertain the hot topics in research, which can provide a future research direction for researchers.

## 2 Methods

### 2.1 Data source and collection

The SCOPUS database was searched on December 27, 2020 to identify articles published from its inception to December 27, 2020. We chose the SCOPUS database because of its accuracy and widespread inclusion of medical articles, which make it suitable for bibliometric analysis.^[16,17]^ The search was performed on 2 occasions. The first search was performed to identify the general treatment trends for facial nerve palsy, using the following search terms: TITLE (“peripheral facial pa∗” OR “facial nerve pa∗” OR “bell's palsy” OR “Ramsay-Hunt Syndrome” OR “facial nerve injury”) AND ABS (treat∗ OR therapy OR intervention OR medication) (Search 1). In the second search, to focus on the trends of complementary and alternative treatment for facial nerve palsy, we narrowed down the scope of the search, using the following search terms: TITLE (“peripheral facial pa∗” OR “facial nerve pa∗” OR “bell's palsy” OR “Ramsay-Hunt Syndrome” OR “facial nerve injury”) AND ABS (treat∗ OR therapy OR intervention OR medication) AND ALL (integrative OR complement∗ OR alternative OR tradition∗) (Search 2). The search was limited to “TITLE” to exclude articles with low relevance. All articles identified in these searches were included in the analysis without article type or language restrictions. Ultimately, 1609 articles were identified in Search 1 and 223 in Search 2. All of these articles were included in the subsequent analysis.

### 2.2 Data analysis and visualization

Information on the articles, such as year, research area, journal title, country, institution, authors, and basic statistics was derived using the intrinsic function of SCOPUS.

The identified articles were imported to the VOS viewer 1.6.16 (Nees Jan van Eck and Ludo Waltman, Leiden University, Leiden, The Netherlands), a software useful for bibliometric mapping,.^[18]^ to conduct a co-occurrence analysis. All terms that met the minimum number of occurrences were included in the analysis without restrictions on relevance score. As a normalization method, “association strength” was applied, and the minimum cluster size was set to 30. In the co-occurrence network map, the terms were expressed as nodes, with higher frequency terms having a larger node size.^[19]^ Nodes that were close to each other indicated high relevance, and lines connecting nodes indicated that the terms co-occurred.^[20]^

Before full-scale analysis, terms related to databases such as “China National Knowledge Infrastructure (CNKI),” “Cochrane library,” and “Latin American and Caribbean Health Sciences Literature (LILACS)” were excluded, as were terms related to the basic structure of articles, such as “study design” and “trial registration,” and terms that were meaningless to the analysis, such as “January,” “February,” and so on. A thesaurus file was created and applied to the analysis so that synonyms could be unified into 1 term. For example, “idiopathic facial paralysis,” “idiopathic peripheral facial paralysis,” “idiopathic facial nerve paralysis,” and “idiopathic facial palsy” were unified as “Bell's palsy.” Full terms and abbreviations were also unified, such as “Ramsay Hunt Syndrome” and “RHS,” “electromyography,” and “EMG,” and so on.

### 2.3 Ethical approval

Ethical approval was not necessary because this study did not involve human or animal research.

## 3 Results

### 3.1 Analysis of publications by year

**Figure 1:**
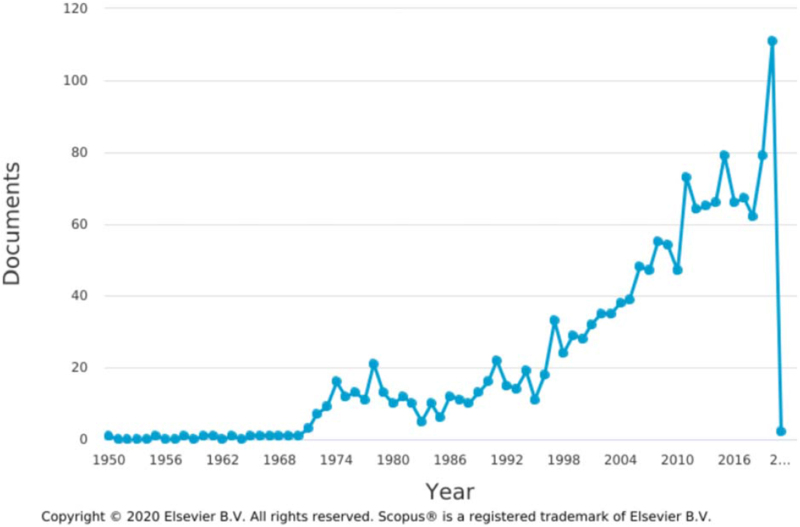
Annual publication trend of research into facial nerve palsy treatment in Search 1, conducted from inception to December 27, 2020.

**Figure 2:**
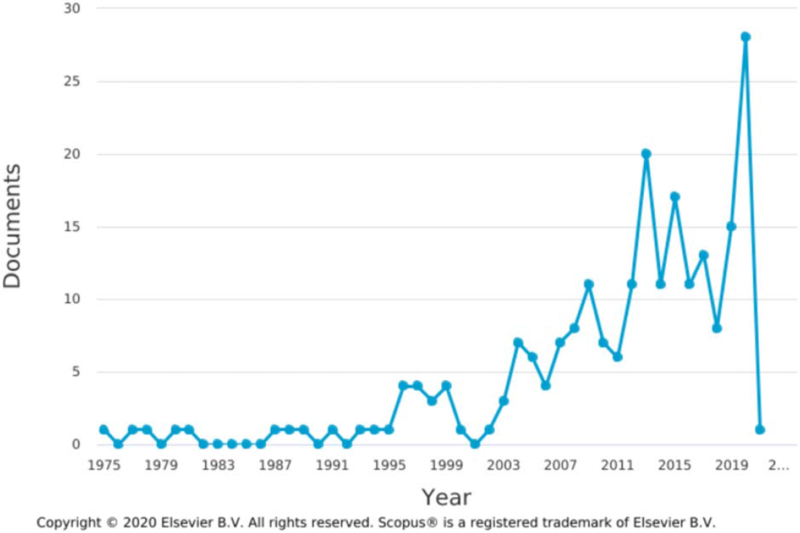
Annual publication trend of research into facial nerve palsy treatment in Search 2, conducted from inception to December 27, 2020.

A total of 1609 and 223 articles were obtained in Searches 1 and 2, respectively. An article published in 1950 was the earliest in Search 1, while an article published in 1975 was the earliest in Search 2. Although there were some variations, particularly in Search 2, the number of articles published showed an overall tendency to increase annually, and the largest number of articles was published in 2020, with 111 and 28 in Searches 1 and 2, respectively (Figs. 1 and 2).

### 3.2 Analysis of research areas and journals

Table 1 shows the top 10 most common subject areas and journals of the 1609 articles in Search 1. In terms of subject area, “medicine” was overwhelmingly the most common (1522 articles; 77.6%), followed by “neuroscience” (143; 7.3%), “biochemistry, genetics, and molecular biology” (56; 2.9%), “health professions” (51; 2.6%) and “dentistry” (48; 2.4%). In terms of the journals represented, “Otology and Neurotology” (48; 3.0%) produced most articles, followed by “Practical Oto-Rhino-Laryngologica”(43; 2.7%), “Laryngoscope” (40; 2.5%), “Chinese Acupuncture & Moxibustion” (37; 2.3%), and “Acta Oto-Laryngologica” (33; 2.1%).

**Table 1:** Most common subject areas and journals in Search 1.

Rank	Subject Area	Articles (n), % of All Articles (1609)	Rank	Journal Title	Articles (n), % of all articles (1609)
1	Medicine	n = 1522, 77.6%	1	*Otology and Neurotology*	n = 48, 3.0%
2	Neuroscience	n = 143, 7.3%	2	*Practica Oto-Rhino-Laryngologica*	n = 43, 2.7%
3	Biochemistry, Genetics and Molecular Biology	n = 56, 2.9%	3	*Laryngoscope*	n = 40, 2.5%
4	Health Professions	n = 51, 2.6%	4	*Chinese acupuncture & moxibustion*	n = 37, 2.3%
5	Dentistry	n = 48, 2.4%	5	*Acta Oto-Laryngologica*	n = 33, 2.1%
6	Pharmacology, Toxicology and Pharmaceutics	n = 25, 1.3%	6	*Practica Otologica*	n = 23, 1.4%
7	Engineering	n = 22, 1.1%	7	*Journal of Laryngology and Otology*	n = 18, 1.1%
8	Immunology and Microbiology	n = 17, 0.9%	8	*Otolaryngology - Head and Neck Surgery*	n = 14, 0.9%
9	Nursing	n = 12, 0.6%	9	*International Journal of Pediatric Otorhinolaryngology*	n = 13, 0.8%
10	Social Sciences	n = 11, 0.5%	10	*American Journal of Otology*	n = 12, 0.7%

### 3.3 Analysis of countries, institutions, and authors

#### 3.3.1 Countries

With the exception of undefined countries, 81 countries were identified in Search 1 and 43 in Search 2. Among the 81 countries in Search 1, the United States published the largest number of articles, with 268 (16.7%), followed by Japan (214; 13.3%) and China (190; 11.8%). Among the 43 countries in Search 2, China published the largest number of articles, with 86 (38.6%), followed by the United States (39; 17.5%) and South Korea (16; 7.1%). Although there were differences in the exact rankings of the top 11 countries between the searches, 10 of the top 11 countries were the same. The most productive countries are listed in Tables 2 and 3.

**Table 2:** Most productive countries in Search 1.

	Country	Frequency (n)	% (of 1609)
1	United States	268	16.7
2	Japan	214	13.3
3	China	190	11.8
4	United Kingdom	87	5.4
5	South Korea	73	4.5
6	Turkey	70	4.4
7	Germany	67	4.2
8	India	56	3.5
9	Italy	48	3.0
10	Brazil	40	2.5
11	Canada	40	2.5

**Table 3:** Most productive countries in Search 2.

	Country	Frequency	% (of 223)
1	China	86	38.6
2	United States	39	17.5
3	South Korea	16	7.2
4	Italy	9	4.0
5	Turkey	9	4.0
6	United Kingdom	9	4.0
7	Japan	7	3.1
8	Canada	6	2.7
9	India	5	2.2
10	Brazil	4	1.8
11	Taiwan	4	1.8

#### 3.3.2 Institutions

In Searches 1 and 2, 160 and 21 institutions published >3 articles, respectively. In Search 1, among 160 institutions, Kyung Hee University published the largest number of articles (25; 1.6%), followed by Yamagata University Faculty of Medicine (17; 1.1%) and Sichuan University (14; 0.9%). In Search 2, among the 21 institutions, Chengdu University of Traditional Chinese Medicine published the largest number of articles (11; 4.9%), followed by Sichuan University (9; 4.0%) and Kyung Hee University (7; 3.1%). Although there were changes in the top 13institutions per search, the same 7 were ranked in the top 13 in both searches. The most prolific institutions are shown in Tables 4 and 5.

**Table 4:** Most productive institutions in Search 1.

	Institutions	Frequency (n)	% (of 1609)
1	Kyung Hee University	25	1.6
2	Yamagata University Faculty of Medicine	17	1.1
3	Sichuan University	14	0.9
4	Chengdu University of Traditional Chinese Medicine	13	0.8
5	Ehime University School of Medicine	13	0.8
6	University of Toronto	13	0.8
7	Harvard Medical School	12	0.7
8	Sapienza University of Rome	12	0.7
9	Amsterdam UMC - University of Amsterdam	11	0.7
10	University of Dundee	11	0.7
11	Capital Medical University	11	0.7
12	Radboud University Nijmegen Medical Centre	10	0.6
13	West China School of Medicine/West China Hospital of Sichuan University	10	0.6

**Table 5:** Most productive institutions in Search 2.

	Institutions	Frequency (n)	% (of 223)
1	Chengdu University of Traditional Chinese Medicine	11	4.9
2	Sichuan University	9	4.0
3	Kyung Hee University	7	3.1
4	Harvard Medical School	6	2.7
5	Anhui University of Chinese Medicine	5	2.2
6	University of Michigan, Ann Arbor	5	2.2
7	Sichuan Provincial People's Hospital	5	2.2
8	West China School of Medicine/West China Hospital of Sichuan University	5	2.2
9	Fudan University	4	1.8
10	Shanghai University of Traditional Chinese Medicine	4	1.8
11	Capital Medical University	4	1.8
12	Sapienza University of Rome	4	1.8
13	General Hospital of People's Liberation Army	4	1.8

#### 3.3.3 Authors

In Searches 1 and 2, 159 and 14 authors published more than three articles, respectively. Among the 159 authors in Search 1, Yeo SG published the largest number of articles, with 16 (1.0%), followed by Aoyagi M (14; 0.9%). Among the 14 authors in Search 2, Li C, Li Y, and Zhou D published the largest number of articles, with five each (2.2%). The most prolific authors are listed in Tables 6 and 7.

**Table 6:** Most productive authors in Search 1.

	Authors	Frequency (n)	% (of 1609)
1	Yeo SG (Kyung Hee University, South Korea)	16	1.0
2	Aoyagi M (Yamagata University, Japan)	14	0.9
3	Guntinas-Lichius O (Jena University Hospital, Germany)	13	0.8
4	Hato N (Ehime University School of Medicine, Japan)	13	0.8
5	Murakami S (Nagoya City University, Japan)	13	0.8
6	Yanagihara N (Ehime University School of Medicine, Japan)	13	0.8
7	Daly F (Frontier Science (Scotland) Ltd, United Kingdom)	12	0.7
8	Byun JY (Kyung Hee University, South Korea)	11	0.7
9	Inamura H (Yamagata University, Japan)	11	0.7
10	Park MS (Kyung Hee University, South Korea)	11	0.7

**Table 7:** Most productive authors in Search 2.

	Authors	Frequency (n)	% (of 223)
1	Li C (Anhui University of Chinese Medicine, China)	5	2.2
2	Li Y (Chengdu University of Traditional Chinese Medicine, China)	5	2.2
3	Zhou D. (Sichuan University, China)	5	2.2
4	Li N (Sichuan University, China)	4	1.8
5	Wu H (Anhui University of Chinese Medicine, China)	4	1.8
6	Yang J (First Affiliated Hospital of Anhui University of Chinese Medicine, China)	4	1.8
7	He, L (Sichuan University, China)	3	1.3
8	Jun HK (Chungnam National University, South Korea)	3	1.3
9	Kan H (Anhui University of Chinese Medicine, China)	3	1.3
10	Kim DH (Chungnam National University, South Korea)	3	1.3
11	Li Y (Chengdu University of Traditional Chinese Medicine, China)	3	1.3
12	Li Y (Sichuan Provincial People's Hospital, China)	3	1.3
13	Xu C (Anhui University of Chinese Medicine, China)	3	1.3

### 3.4 Keywords

In the present study; terms that met a minimum number of occurrences threshold were defined as keywords and used to identify research hotspots in the treatment of facial nerve palsy in each analysis.^[21]^

#### 3.4.1 Keywords in Search 1

**Figure 3:**
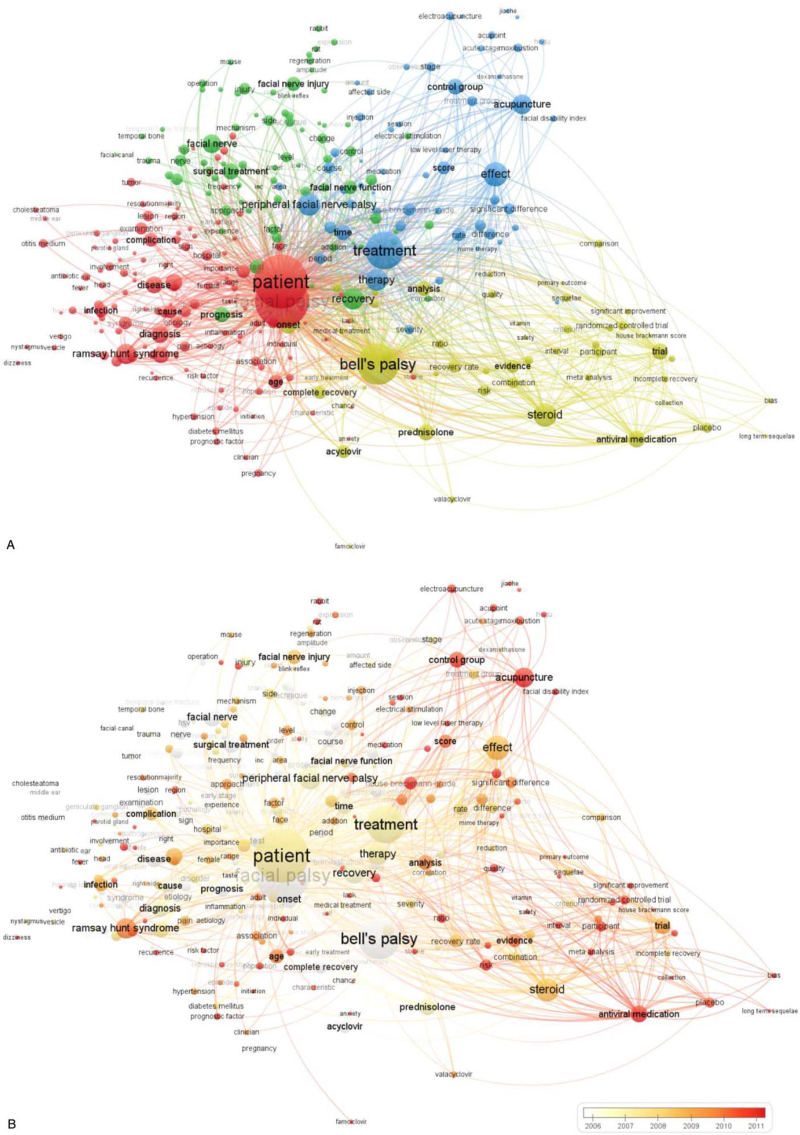
Co-occurrence analysis of 316 keywords from the title and abstract in 1609 articles. (A) Mapping of keywords in research into facial nerve palsy treatment; 316 keywords are divided into 4 clusters. (B) Overlaid visualization map by average publication year, with white representing earlier and red representing later.

Terms with at least 20 occurrences in the titles and abstracts of the articles were defined as keywords, resulting in 316 extractions. The co-occurrence of these 316 terms was analyzed using the VOS viewer (Fig. 3A). The keywords were then classified into 4 clusters, indicating that research into the treatment of facial nerve palsy can be categorized into 4 main categories: “pathophysiology” (cluster 1, red), “surgical treatment” (cluster 2, green), “complementary and alternative treatment” (cluster 3, blue), and “medication treatment” (cluster 4, yellowish green). A total of 131 items were included in the “pathophysiology” cluster, with the most frequent keywords being “patient” (4264 times), “facial palsy” (4258 times), “Ramsay Hunt syndrome” (609 times), “diagnosis,” (455 times) and “disease” (424 times). Eighty items were included in the “surgical treatment” cluster, with the most frequent keywords being “recovery” (706 times), “facial nerve” (527 times), “surgical treatment” (396 times), “prognosis” (359 times), and “facial nerve injury” (306 times). Fifty-six items were included in the “complementary and alternative treatment” cluster, with the most frequent keywords being “treatment” (2129 times), “effect” (878 times), “peripheral facial nerve palsy” (728 times), “therapy” (711 times), and “acupuncture” (578 times). Forty-nine items were included in the “medication treatment” cluster, with the most frequent keywords being “Bell's palsy” (2563 times), “steroid” (814 times), “onset” (481 times), “antiviral medication” (414 times), and “prednisolone” (391 times).

Figure 3B shows the same co-occurrence map overlaid with different colors depending on the average publication year of the keyword, with white representing earlier publication and red representing more recent publication. Overall, “complementary and alternative treatment” and “medication treatment” were the latest research trends.

To focus more on treatment, another search was carried out to identify terms related to treatment that appeared >20 times in the articles’ keywords provided by the authors. As a result, thirty items were derived (see Table, Supplemental Digital Content 1, which illustrates the occurrence, average publication year, and average citation count of 30 keywords that met the minimum occurrence number of 20 in Search1). The top 5 terms were “acyclovir” (235 times), “prednisolone” (211 times), “antiviral agent” (190 times), “prednisone” (161 times), and “steroid” (161 times). Based on the average publication year, the 5 most recent terms were “famciclovir” (2013.04), “electroacupuncture” (2011.23), “moxibustion” (2011.04), “glucocorticoid” (2010.79), “methylprednisolone” (2010.63), whereas the 5 earliest terms were “corticotrophin” (1980.30), “cortisone” (1994.71), “electric stimulation therapy” (2001.36), “hydrocortisone” (2003.64), “prednisone” (2003.78). Based on average citation counts, the 5 most common terms were “massage” (41.22), “drug combination” (30.22), “anti-inflammatory agents” (29.71), “cortisone” (26.62), and “prednisone” (26.13), whereas and the 5 least common terms were “electroacupuncture” (2.74), “conservative treatment” (6.12), “acupuncture” (7.09), “moxibustion” (7.92), and “antibiotic agent” (8.27).

**Figure 4:**
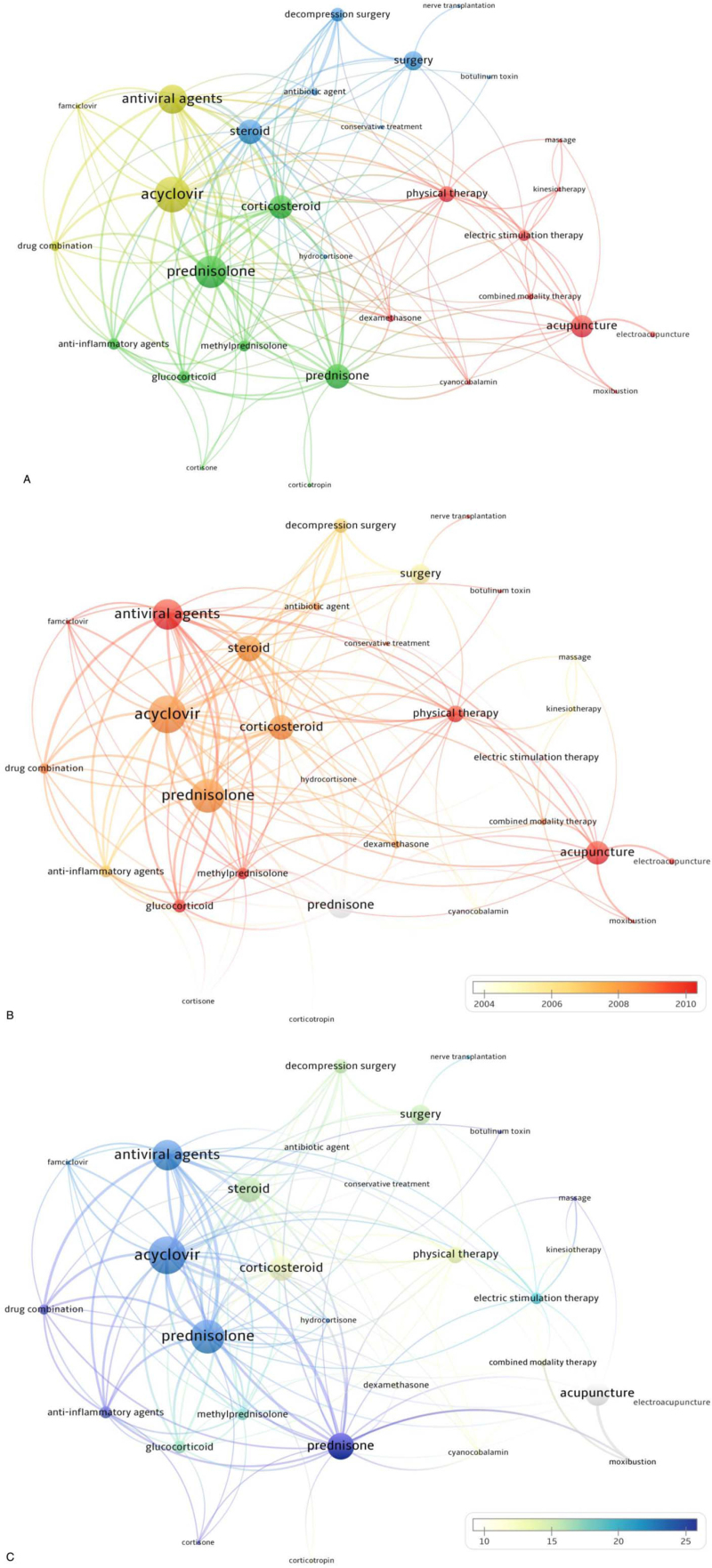
Co-occurrence analysis of 30 keywords from article's keywords in 1609 articles. (A) Mapping of keywords; 30 keywords are divided into 4 clusters. (B) Overlaid visualization map by average publication year, with white representing earlier and red representing later. (C) Overlaid visualization map by average citation count, with blue representing more citations and white representing less.

We also created a co-occurrence network map of 30 items using VOS viewer (Fig. 4A). The map was classified into 4 clusters, indicating the 4 main categories of research into facial nerve palsy treatment more clearly than the previous results: “steroids,” “antivirals,” “surgical treatment,” and “complementary and alternative treatment.”

In Figure 4B an overlay visualization shows the average publication year of the keyword, with white representing earlier and red representing more recent publication. Overall, “antivirals” and “complementary and alternative treatment” tended to be more recent.

The overlay visualization in Figure 4C shows the average citation counts, with blue representing more citation counts and white representing fewer citation counts. Overall, “steroids” and “antivirals” showed the most citation counts.

#### 3.4.2 Keywords in Search 2

Terms related to treatment that appeared >2 times in the articles’ keywords among the 223 articles in Search 2 were extracted. As a result, 32 items were derived (see Table, Supplemental Digital Content 2, which illustrates the occurrence, average publication year, and average citation count of 32 keywords that met the minimum occurrence number of 2 in Search 2). These 32 keywords were divided into 4 main categories: “nutrient,” “traditional Chinese medicine,” “rehabilitation,” and “eye care.” Moreover, the specific elements of each category could be discerned, for example, “thiamine,” “ascorbic acid,” and “vitamin B” in the “nutrient” category, “herbal medicine,” “laser acupuncture” and “thread embedding acupuncture” in the “traditional Chinese medicine” category, “low-level laser therapy” and “biofeedback” in the “rehabilitation” category, and “eye protection” and “tarsorrhaphy” in the “eye care” category.

**Figure 5:**
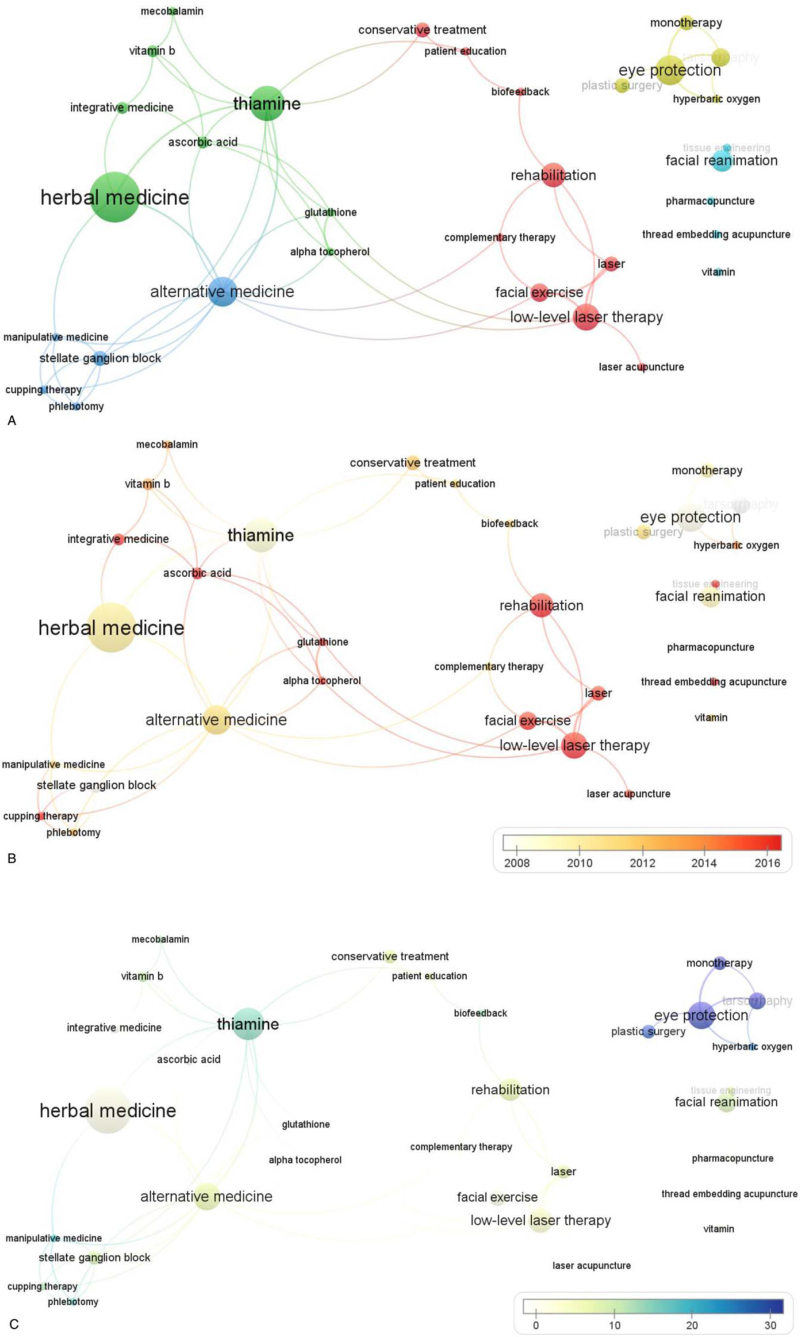
Co-occurrence analysis of 32 keywords from articles’ keywords in 223 articles. (A) Mapping of keywords; 32 keywords are divided into 5 clusters. (B) Overlaid visualization map by average publication year, with white representing earlier and red representing later. (C) Overlaid visualization map by average citation count, with blue representing more citations and white representing less.

We also created a co-occurrence network map of the 32 items using VOS viewer (Fig. 5A). In overlay visualizations by average publication year and average citation count (Fig. 5B and C). There was no noticeable tendency in average publication year among the 4 categories; overall, “eye care” showed the most citations.

## 4 Discussion

Medication treatments, including steroids and antivirals, can be applied during the acute period of facial nerve palsy, and several of the treatments that are subsequently administered to promote recovery are complementary and alternative treatment.^[22,23]^

This study comprised a bibliometric and visualized analysis to identify research trends in facial nerve palsy treatment. Over time, research has shown an increasing tendency, with the largest number of articles published in 2020 in both searches. Nonetheless, more research will be needed to ensure that that this trend continues in future. The proportion of articles attributed to the top 10 institutions or authors was not so large. With regards to country, the top 3 countries accounted for 41.8% and 63.2% of the total in Searches 1 and 2, respectively. Ten of the top 11 countries were same in both searches, so most research on facial nerve palsy has only been conducted in a few countries.

Based on the number of published articles, “medicine” was the most actively studied subject area. In terms of journals, even the top five journals represented did not have a high proportion of the total, indicating that articles have been published across diffuse journals. However, with the exception of only one, all of the top 10 journals were otolaryngology-related, suggesting that the subject focus of the journal tended to be similar across the articles. Additionally, otolaryngology may have accounted for a large portion of articles in the “medicine” subject area.

In the 1609 articles obtained in Search 1, we conducted co-occurrence analysis twice. The results of the 2 analyses were the same in the large framework, but this approach allowed us to better understand the research trends and hotspots associated with the treatment of facial nerve palsy.

Firstly, through a co-occurrence analysis of the 316 keywords extracted from the titles and abstracts, we discovered 4 major treatment categories: pathophysiology, surgical treatment, complementary and alternative treatment, and medication treatment. In the “pathophysiology” category terms such as “diabetes mellitus,” “pregnancy” and “hypertension” were included, as research has been conducted into the underlying diseases that could affect prognosis in treatment.^[24,25]^ In the “medication treatment” category, we identified the most commonly used drugs for core treatment in clinical practice, as well as terms such as “evidence,” “randomized controlled trial,” “meta analysis,” and “systematic review.” These terms tended to appear relatively recently, indicating that the studies in this category with a fairly high level of evidence were conducted recently.^[5,6,26–28]^ In the “complementary and alternative treatment” category, terms such as “acupuncture,” “rehabilitation,” “electroacupuncture,” “physical therapy,” and “low level laser therapy” were included.^[29–32]^ Moreover, this category showed a tendency towards more recent studies, just as with “medication treatment”. In other words, the research areas on the treatment of facial nerve palsy that are presently being focused on were “medication treatment” and “complementary and alternative treatment,” indicating the probable direction of future research.

With a second analysis of 30 keywords extracted from the author keywords, the 4 main areas of research into the treatment of facial nerve palsy were more clearly identified: “steroids,” “antivirals,” “surgical treatment,” and “complementary and alternative treatment.”

Later average publication years were probably associated with lower average citation counts. However, the most recent keyword, “famciclovir” (2013.04) had a relatively high citation count (22.00), whereas “prednisolone” (2008.45) had a large number of citations (22.86) in a relatively short period of time. Meanwhile, “electroacupuncture” (2011.23) had a citation count of 2.74 and “acupuncture” (2010.49) a citation count of 7.09, which rank among lower numbers of citations. Taken together, low citation count likely indicates that the study quality was relatively poor, and continuous research will be necessary in areas that have recently been studied but do not yet have sufficient qualitative evidence such as “complementary and alternative treatment”.^[12,33,34]^

In the second search, to find out more about the trends in complementary and alternative treatment, we narrowed the scope of the search, with the minimum occurrence number being lowered to 2 to extract even small frequency keywords. When analyzing 32 keywords, the average publication year was more recent, while the average citation count was low. For example, the citation count of “thread-embedding acupuncture” (2019.50) was 0.00, while that of “ascorbic acid” (2016.67) was 0.33, that of “laser acupuncture” (2015.50) was 1.50 and that of “low-level laser therapy” (2018.25) was 4.75. As mentioned earlier, further research is necessary to improve the quality of the studies and to establish a basis for these treatments.^[32,35,36]^

The present study had several limitations. The article search was only conducted in one database, and because only a fraction of the data were extracted, namely country, institution, author, and keyword, the main trends could be identified. However, these did not fully reflect the full substance of the present research. In addition, subjective perspectives of authors were involved, such as setting the minimum number of occurrences in the process of extracting keywords, setting counting method, and minimizing cluster size in the process of organizing the network map.

Nevertheless, it is meaningful that the study identified research trends in the treatment of facial nerve palsy and direction to future research including the latest articles.

## 5 Conclusion

The present study investigated the overall trends of facial nerve palsy treatment using bibliometric and visualized analyses. In particular, the areas focused on in research so far were determined using analysis of keywords. To date, research on medication treatment has been the main focus, and antiviral use among medication treatment and complementary and alternative treatment has emerged in recent years. However, research on complementary and alternative treatment is still lacking, and the quality of the research is relatively low compared to that in research on medication treatment.^[12,34]^

Medication treatment, including steroids and antivirals, can be applied during the acute period of facial nerve palsy, and many of the treatments that are subsequently administered are complementary and alternative treatments.^[22,23,37]^ Considering that 30% of patients with facial nerve palsy have sequelae that reduce their quality of life and cause psychological problems,^[38]^ any treatment additional to medication that can promote patient recovery is important. It follows that research into medication, which is the hotspot of present research trends, needs to continue, whereas more efforts should be made to conduct high-quality research into complementary and alternative treatment.

## Author contributions

**H-NK:** Writing-Original Draft, Investigation, Data Curation; **J-HK:** Investigation, Data Curation; **BG:** Conceptualization, Methodology, Formal analysis, Investigation, Writing-Review & Editing; **S-SN:** Writing-Review & Editing, Supervision, Project administration, Funding acquisition. All authors approved the final manuscript.

**Conceptualization:** Bonhyuk Goo.

**Data curation:** Ha-Na Kim, Jung-Hyun Kim.

**Formal analysis:** Bonhyuk Goo.

**Funding acquisition:** Sang-Soo Nam.

**Investigation:** Ha-Na Kim, Jung-Hyun Kim, Bonhyuk Goo.

**Methodology:** Bonhyuk Goo.

**Project administration:** Sang-Soo Nam.

**Supervision:** Sang-Soo Nam.

**Writing – original draft:** Ha-Na Kim.

**Writing – review & editing:** Bonhyuk Goo, Sang-Soo Nam.

## Supplementary Material

Supplemental Digital Content

## Supplementary Material

Supplemental Digital Content
