# Supplementary material for: A bibliometric analysis of research on the treatment of facial nerve palsy
Source: Medicine (Baltimore). 2021 Aug 20;100(33):e26984. doi: 10.1097/MD.0000000000026984 (PMC8376370; doi:10.1097/MD.0000000000026984)
Supplement: Supplemental Digital Content [file medi-100-e26984-s001.doc]

Supplemental Digital Content 1. Table that illustrates the occurrence, average publication year, and average citation count of 30 keywords that met the minimum occurrence number of 20 in Search 1

| Rank | Keywords | Occurrences | Average published year | Average citation count |
| --- | --- | --- | --- | --- |
| 1 | Acyclovir | 235 | 2008.46 | 23.00 |
| 2 | Prednisolone | 211 | 2008.45 | 22.86 |
| 3 | Antiviral agent | 190 | 2010.54 | 22.76 |
| 4 | Prednisone | 161 | 2003.78 | 26.13 |
| 5 | Steroid | 161 | 2008.22 | 15.73 |
| 6 | Corticosteroid | 154 | 2008.43 | 12.66 |
| 7 | Acupuncture | 146 | 2010.49 | 7.09 |
| 8 | Surgery | 126 | 2005.60 | 15.68 |
| 9 | Physical therapy | 105 | 2009.69 | 13.63 |
| 10 | Decompression surgery | 91 | 2006.82 | 15.62 |
| 11 | Glucocorticoid | 81 | 2010.79 | 16.98 |
| 12 | Anti-inflammatory agents | 75 | 2007.09 | 29.71 |
| 13 | Electric stimulation therapy | 73 | 2001.36 | 19.37 |
| 14 | Methylprednisolone | 71 | 2010.63 | 17.76 |
| 15 | Drug combination | 63 | 2008.68 | 30.22 |
| 16 | Antibiotic agent | 49 | 2008.51 | 8.27 |
| 17 | Dexamethasone | 48 | 2008.21 | 10.90 |
| 18 | Combined modality therapy | 42 | 2008.74 | 11.60 |
| 19 | Electroacupuncture | 39 | 2011.23 | 2.74 |
| 20 | Cyanocobalamin | 31 | 2004.94 | 13.13 |
| 21 | Famciclovir | 28 | 2013.04 | 22.00 |
| 22 | Kinesiotherapy | 28 | 2006.39 | 12.89 |
| 23 | Conservative treatment | 25 | 2009.20 | 6.12 |
| 24 | Hydrocortisone | 25 | 2003.64 | 22.08 |
| 25 | Moxibustion | 24 | 2011.04 | 7.92 |
| 26 | Botulinum toxin | 23 | 2010.00 | 25.39 |
| 27 | Massage | 23 | 2006.43 | 41.22 |
| 28 | Cortisone | 21 | 1994.71 | 26.62 |
| 29 | Nerve transplantation | 21 | 2010.05 | 20.24 |
| 30 | Corticotrophin | 20 | 1980.30 | 12.40 |
