# Supplementary material for: A bibliometric analysis of research on the treatment of facial nerve palsy
Source: Medicine (Baltimore). 2021 Aug 20;100(33):e26984. doi: 10.1097/MD.0000000000026984 (PMC8376370; doi:10.1097/MD.0000000000026984)
Supplement: Supplemental Digital Content [file medi-100-e26984-s002.doc]

Supplemental Digital Content 2. Table that illustrates the occurrence, average publication year, and average citation count of 32 keywords that met the minimum occurrence number of two in Search 2

| Rank | Keywords | Occurrences | Average published year | Average citation count |
| --- | --- | --- | --- | --- |
| 1 | Herbal medicine | 17 | 2010.41 | 2.24 |
| 2 | Thiamine | 11 | 2008.91 | 14.55 |
| 3 | Alternative medicine | 9 | 2011.22 | 7.44 |
| 4 | Eye protection | 9 | 2008.44 | 57.44 |
| 5 | Low-level laser therapy | 8 | 2018.25 | 4.75 |
| 6 | Rehabilitation | 7 | 2016.43 | 8.14 |
| 7 | Facial reanimation | 6 | 2009.67 | 9.83 |
| 8 | Facial exercise | 5 | 2015.60 | 2.00 |
| 9 | Tarsorrhaphy | 5 | 2004.20 | 30.00 |
| 10 | Laser | 4 | 2015.50 | 8.25 |
| 11 | Stellate ganglion block | 4 | 2006.25 | 9.25 |
| 12 | Monotherapy | 4 | 2010.00 | 110.25 |
| 13 | Conservative treatment | 4 | 2012.25 | 7.75 |
| 14 | Plastic surgery | 4 | 2011.25 | 27.75 |
| 15 | Ascorbic acid | 3 | 2016.67 | 0.33 |
| 16 | Vitamin b | 3 | 2013.33 | 10.67 |
| 17 | Integrative medicine | 3 | 2018.67 | 0.67 |
| 18 | Alpha tocopherol | 2 | 2015.50 | 0.50 |
| 19 | Glutathione | 2 | 2015.50 | 0.50 |
| 20 | Manipulative medicine | 2 | 2012.00 | 18.50 |
| 21 | Cupping therapy | 2 | 2016.00 | 12.50 |
| 22 | Phlebotomy | 2 | 2012.50 | 16.00 |
| 23 | Complementary therapy | 2 | 2012.00 | 2.50 |
| 24 | Hyperbaric oxygen | 2 | 2014.00 | 24.50 |
| 25 | Patient education | 2 | 2012.00 | 9.00 |
| 26 | Biofeedback | 2 | 2011.50 | 13.00 |
| 27 | Mecobalamin | 2 | 2013.50 | 12.50 |
| 28 | Laser acupuncture | 2 | 2015.50 | 1.50 |
| 29 | Tissue engineering | 2 | 2016.50 | 8.00 |
| 30 | Pharmacopuncture | 2 | 2007.50 | 2.00 |
| 31 | Thread embedding acupuncture | 2 | 2019.50 | 0.00 |
| 32 | Vitamin | 2 | 2011.50 | 2.00 |
